# Crystal structure and Hirshfeld surface analysis of ethyl (*E*)-4-[(4-hy­droxy-3-meth­oxy-5-nitro­benzyl­idene)amino]­benzoate

**DOI:** 10.1107/S2056989018009465

**Published:** 2018-07-10

**Authors:** Antony Samy Victoria, Palaniyappan Sivajeyanthi, Natarajan Elangovan, Kasthuri Balasubramani, Thathan Kolochi, Kanagasabapathy Thanikasalam

**Affiliations:** a PG & Research Department of Chemistry, Government Arts College (Autonomous), Arignar Anna Government Arts College, Musiri 621 211, Tamil Nadu, India; b Department of Chemistry, Government Arts College (Autonomous), Thanthonimalai, Karur 639 005, Tamil Nadu, India; c Department of Chemistry, Periyar EVR College (Autonomous), Thiruchirapalli 620 023, Tamil Nadu, India

**Keywords:** crystal structure, Schiff base, Hirshfeld surface analysis, hydrogen bonding

## Abstract

The title Schiff base compound, C_17_H_16_N_2_O_6_, has an *E* configuration with respect to the C=N bond, with a dihedral angle between the two benzene rings of 31.90 (12)°. There is an intra­molecular O—H⋯O_nitro_ hydrogen bond present forming an *S*(6) ring motif. In the crystal, mol­ecules are linked by pairs of O—H⋯O hydrogen bonds, forming inversion dimers enclosing an *R*_2_^2^(4) ring motif. The dimers are linked about an inversion centre by pairs of C—H⋯O hydrogen bonds, which enclose *R*_2_^2^(22) loops, forming chains propagating along the [10] direction. Hirshfeld surface analysis and fingerprint plots show enrichment ratios for the H⋯H, O⋯H and C⋯H contacts, indicating a high propensity of such inter­actions in the crystal. Both the nitro group and the CH_3_–CH_2_–O– group are positionally disordered.

## Abstract 2

The title Schiff base compound displays a *trans* configuration with respect to the C=N bond, with the two benzene rings being inclined to each other by 31.90 (12)°.

## Chemical context   

Schiff bases are an important class of compounds in the medicinal and pharmaceutical fields. They play a role in the development of coordination chemistry as they readily form stable complexes with most transition metals. These complexes show inter­esting properties, for *e.g*. their ability to reversibly bind oxygen, catalytic activity in hydrogenation of olefins and transfer of an amino group, photochromic properties, and complexing ability towards toxic metals (Karthikeyan *et al.*, 2006 ▸; Khattab, 2005 ▸; Küçükgüzel *et al.*, 2006 ▸). Recently, hydrazone Schiff base compounds (Cao, 2009 ▸; Zhou & Yang, 2010 ▸; Zhang *et al.*, 2009 ▸) derived from the reaction of aldehydes with hydrazines have been shown to possess excellent biological activities, such as anti-bacterial, anti-convulsant, and anti­tubercular (Bernhardt *et al.*, 2005 ▸; Armstrong *et al.*, 2003 ▸). Herein, we report on the synthesis and crystal structure of the title Schiff base title compound, (*E*)-4-[(4-hy­droxy-3-meth­oxy-5-nitro­benzyl­idene)amino]­benzoate. The Hirshfeld surface analysis was performed in order to visualize, explore and qu­antify the inter­molecular inter­actions in the crystal lattice of the title compound.

## Structural commentary   

**Figure 1:**
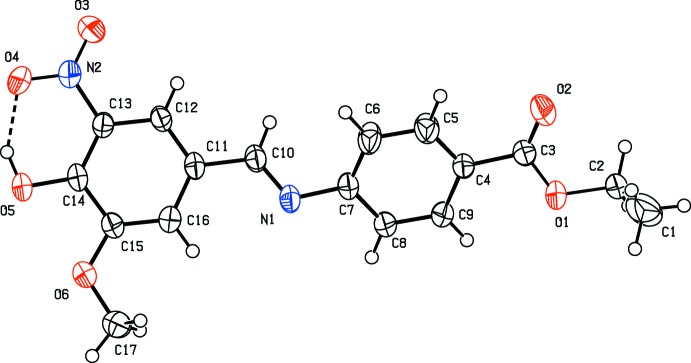
A view of the mol­ecular structure of the title compound, with the atom labelling. Displacement ellipsoids are drawn at the 50% probability level. The intra­molecular O—H⋯O hydrogen bond (Table 1 ▸) is shown as a dashed line. Only the major components of the disordered atoms (O3, O4, C1, C2 and O1) are shown.

The mol­ecular structure of the title Schiff base compound is illustrated in Fig. 1 ▸. The mol­ecule has a *trans* or *E* configuration with respect to the C10=N1 double bond. The dihedral angle between the two benzene rings is 31.90 (12)°. The C10=N1 bond length of 1.267 (3) Å confirms the azomethine bond formation. There is an intra­molecular O—H⋯O hydrogen bond present involving the adjacent hydroxyl and nitro substituents on the C11–C16 benzene ring, forming an *S*(6) ring motif (Fig. 1 ▸ and Table 1 ▸).
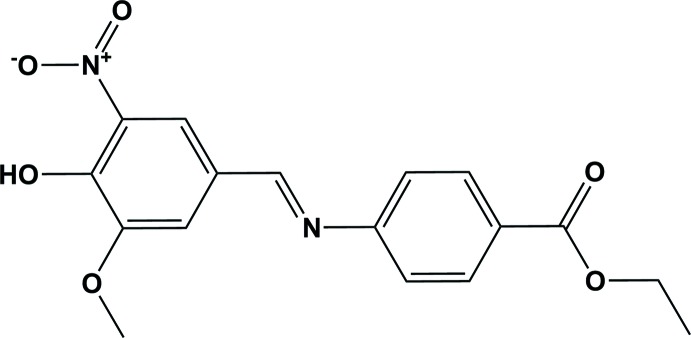


## Supra­molecular features   

**Figure 2:**
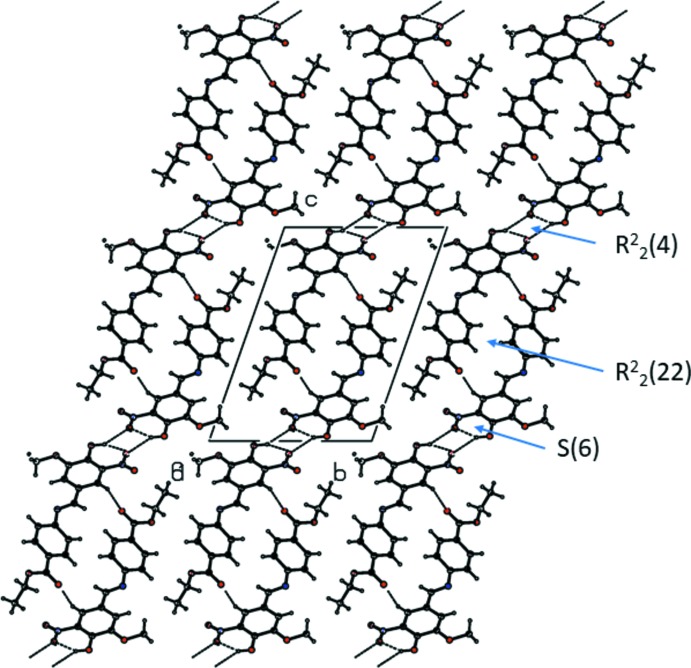
Crystal packing of the title compound, viewed along the *a* axis. The O—H⋯O and C—H⋯O hydrogen bonds (see Table 1 ▸) are shown as dashed lines. Only the major components of the disordered atoms (O3, O4, C1, C2 and O1) are shown.

In the crystal, mol­ecules are linked by pairs of O—H⋯O hydrogen bonds, forming inversion dimers (Table 1 ▸ and Fig. 2 ▸). The dimers are linked by pairs of C—H⋯O hydrogen bonds, so forming chains propagating along [10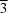]. Within the chains there are two ring motifs present, *viz. R*_2_^2^(4) and 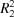
(22), as illustrated in Fig. 2 ▸.

## Database survey   

A search of the Cambridge Structural Database (CSD, Version 5.39, update May 2018; Groom *et al.*, 2016 ▸) for ethyl-4-(benzyl­idene­amino)­benzoate yielded five hits, while a search for the 2-meth­oxy-4-[(phenyl­imino)­meth­yl]phenol skelton gave 25 hits. The most significant structure among these results is that of ethyl-4-[(4-hy­droxy-3-meth­oxy­benzyl­idene)amino]­benzoate (APAMUB; Ling *et al.*, 2016 ▸). The only difference between APAMUB and the title compound is the presence of a nitro group in the title compound. The two benzene rings in APAMUB are inclined to each other by 24.58 (8)° compared to 31.90 (12)° in the title compound. The crystal packing of the two compounds is significantly different. In APAMUB, mol­ecules are linked by O—H⋯N hydrogen bonds, forming chains along [010]. The chains are linked by C—H⋯π and offset π–π inter­actions, resulting in the formation of layers parallel to (10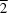). In the title compound there are only O—H⋯O and C—H⋯O hydrogen bonds present; no C—H⋯π nor offset π–π inter­actions are present.

## Hirshfeld surface analysis   

**Figure 3:**
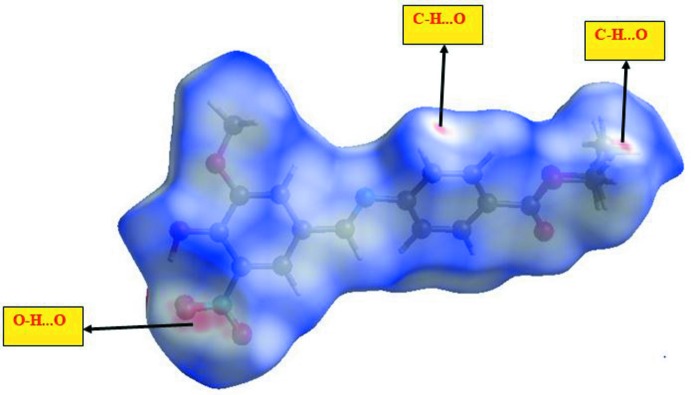
Hirshfeld surfaces mapped over *d*_norm_ for the title compound.

**Figure 4:**
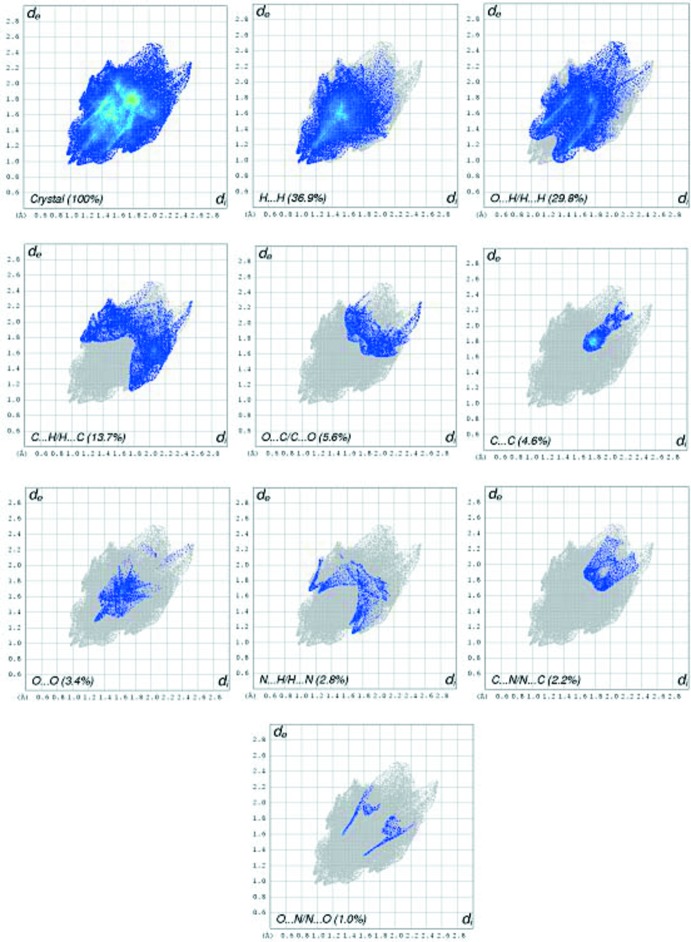
2D fingerprint plots and relative contributions of the atom pairs to the Hirshfeld surface of the title compound.

Hirshfeld surfaces and their associated two-dimensional (2D) fingerprint plots (Soman *et al.*, 2014 ▸) have been used to quantify the various inter­molecular inter­actions in the title compound. The Hirshfeld surface of a mol­ecule is mapped using the descriptor *d*_norm_, which encompasses two factors: one is *d*_e_, representing the distance of any surface point nearest to the inter­nal atoms; another one is *d*_i_, representing the distance of the surface point nearest to the exterior atoms and also with the van der Waals radii of the atoms (Dalal *et al.*, 2015 ▸). The Hirshfeld surfaces mapped over *d*_norm_ (range of −0.502–1.427 a.u.) are displayed in Fig. 3 ▸. The dominant inter­actions between the oxygen (O) and hydrogen (H) atoms can be observed in the Hirshfeld surface as the red areas in Fig. 4 ▸. Other visible spots in the Hirshfeld surfaces correspond to C⋯H and H⋯H contacts.

The inter­molecular inter­actions of the title compound, strongly evidenced by the 2D fingerprint plots from the Hirshfeld surface, are shown in Fig. 4 ▸. The H⋯H inter­actions (36.9%) are relatively high compared to the other bonding inter­actions of the total Hirshfeld surface area. However, it is lower than the H⋯H inter­actions (47.4%) in the crystal of ethyl-4-[(4-hy­droxy-3-meth­oxy­benzyl­idene)amino]­benzoate (APAMUB; Ling *et al.*, 2016 ▸). The percentage contributions of the other contacts in the title compound to the total Hirshfeld surface are as follows: O⋯H/H⋯O (29.8%), C⋯H/H⋯C (13.7%), N⋯H/H⋯N (2.8%), C⋯N/N⋯C (2.2%), C⋯C (4.6%), C⋯O/O⋯C (5.6%), O⋯N/N⋯O (1.0%). Such a visual analysis for inter­molecular inter­actions is coherent with those indicated by the X-ray diffraction results, with the O⋯H/H⋯O (29.8%) inter­actions being the most significant after the H⋯H inter­actions (36.9%).

## Synthesis and crystallization   

The title compound was synthesized by the reaction of a 1:1 molar ratio of ethyl-4-amino­benzoate (0.151 mg) and 4-hy­droxy-3-meth­oxy-5-nitro­benzaldehyde (0.134 mg) in an acetic acid solution (10 ml). The reaction mixture was refluxed for 6 h. The solid product formed during refluxing was filtered, washed with methanol and dried over anhydrous calcium chloride in a vacuum desiccator (yield 75%, m.p. 505 K). Brown block-like crystals of the title compound were obtained by slow evaporation of a solution in DMSO.

## Refinement   

Crystal data, data collection and structure refinement details are summarized in Table 2 ▸. The hydroxyl H atom was located in a difference-Fourier map and freely refined. The C-bound H atoms were positioned geometrically and refined as riding: C—H = 0.93–0.97 Å with *U*_iso_(H) = 1.5*U*_eq_(C-meth­yl) and 1.2*U*_eq_(C) for other H atoms. Atoms O3 and O4 of the nitro group are disordered with a refined occupancy ratio of O3/O3′ = O4/O4′ = 0.64 (12):0.36 (12). Atoms O1, C2 and C1 of the ethyl benzoate group are also disordered with a refined occupancy ratio of O1/O1′ = C2/C2′ = C1/C1′ = 0.65 (3): 0.35 (3).

## Supplementary Material

Crystal structure: contains datablock(s) I, global. DOI: 10.1107/S2056989018009465/su5450sup1.cif

Structure factors: contains datablock(s) I. DOI: 10.1107/S2056989018009465/su5450Isup3.hkl

CCDC reference: 1852926

Additional supporting information:  crystallographic information; 3D view; checkCIF report

## Figures and Tables

**Table 1:** Hydrogen-bond geometry (Å, °)

*D*—H⋯*A*	*D*—H	H⋯*A*	*D*⋯*A*	*D*—H⋯*A*
O5—H5*A*⋯O4	0.91 (4)	1.73 (4)	2.54 (2)	146 (3)
O5—H5*A*⋯O4^i^	0.91 (4)	2.49 (4)	3.23 (3)	138 (3)
C12—H12⋯O2^ii^	0.93	2.60	3.471 (3)	156

Symmetry codes: (i) 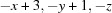; (ii) 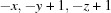.

**Table 2:** Experimental details

Crystal data	
Chemical formula	C_17_H_16_N_2_O_6_
*M*_r_	344.32
Crystal system, space group	Triclinic, *P* 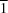
Temperature (K)	296
*a*, *b*, *c* (Å)	4.7565 (3), 11.3377 (9), 15.7590 (13)
α, β, γ (°)	70.415 (4), 87.230 (4), 85.238 (4)
*V* (Å^3^)	797.73 (11)
*Z*	2
Radiation type	Mo *K*α
μ (mm^−1^)	0.11
Crystal size (mm)	0.15 × 0.10 × 0.10

Data collection	
Diffractometer	Bruker Kappa APEXIII CMOS
Absorption correction	Multi-scan (*SADABS*; Bruker, 2016 ▸)
*T*_min_, *T*_max_	0.684, 0.746
No. of measured, independent and observed [*I* > 2σ(*I*)] reflections	27199, 3642, 2484
*R*_int_	0.049
(sin θ/λ)_max_ (Å^−1^)	0.650

Refinement	
*R*[*F* ^2^ > 2σ(*F* ^2^)], *wR*(*F* ^2^), *S*	0.061, 0.176, 1.06
No. of reflections	3642
No. of parameters	279
No. of restraints	148
H-atom treatment	H atoms treated by a mixture of independent and constrained refinement
Δρ_max_, Δρ_min_ (e Å^−3^)	0.30, −0.22

Computer programs: *APEX3*, *SAINT* and *XPREP* (Bruker, 2016 ▸), *SHELXT2014* (Sheldrick, 2015*a* ▸), *SHELXL2014* (Sheldrick, 2015*b* ▸) and *PLATON* (Spek, 2009 ▸).

## supplementary crystallographic information

### Crystal data

**Table:**

C_17_H_16_N_2_O_6_	*Z* = 2
*M**_r_* = 344.32	*F*(000) = 360
Triclinic, *P*1	*D*_x_ = 1.433 Mg m^−^^3^
*a* = 4.7565 (3) Å	Mo *K*α radiation, λ = 0.71073 Å
*b* = 11.3377 (9) Å	Cell parameters from 9528 reflections
*c* = 15.7590 (13) Å	θ = 3.6–27.4°
α = 70.415 (4)°	µ = 0.11 mm^−^^1^
β = 87.230 (4)°	*T* = 296 K
γ = 85.238 (4)°	Block, brown
*V* = 797.73 (11) Å^3^	0.15 × 0.10 × 0.10 mm

### Data collection

**Table:**

Bruker Kappa APEXIII CMOS diffractometer	3642 independent reflections
Radiation source: fine-focus sealed tube	2484 reflections with *I* > 2σ(*I*)
Graphite monochromator	*R*_int_ = 0.049
ω and φ scan	θ_max_ = 27.5°, θ_min_ = 3.8°
Absorption correction: multi-scan (SADABS; Bruker, 2016)	*h* = −6→6
*T*_min_ = 0.684, *T*_max_ = 0.746	*k* = −14→14
27199 measured reflections	*l* = −20→20

### Refinement

**Table:**

Refinement on *F*^2^	Secondary atom site location: difference Fourier map
Least-squares matrix: full	Hydrogen site location: mixed
*R*[*F*^2^ > 2σ(*F*^2^)] = 0.061	H atoms treated by a mixture of independent and constrained refinement
*wR*(*F*^2^) = 0.176	*w* = 1/[σ^2^(*F*_o_^2^) + (0.0654*P*)^2^ + 0.6845*P*] where *P* = (*F*_o_^2^ + 2*F*_c_^2^)/3
*S* = 1.06	(Δ/σ)_max_ = 0.005
3642 reflections	Δρ_max_ = 0.30 e Å^−^^3^
279 parameters	Δρ_min_ = −0.22 e Å^−^^3^
148 restraints	Extinction correction: (SHELXL2018; Sheldrick, 2015b), Fc^*^=kFc[1+0.001xFc^2^λ^3^/sin(2θ)]^-1/4^
Primary atom site location: structure-invariant direct methods	Extinction coefficient: 0.045 (8)

### Special details

**Table:**

Geometry. All esds (except the esd in the dihedral angle between two l.s. planes) are estimated using the full covariance matrix. The cell esds are taken into account individually in the estimation of esds in distances, angles and torsion angles; correlations between esds in cell parameters are only used when they are defined by crystal symmetry. An approximate (isotropic) treatment of cell esds is used for estimating esds involving l.s. planes.

### Fractional atomic coordinates and isotropic or equivalent isotropic displacement parameters (Å^2^)

**Table:**

	*x*	*y*	*z*	*U*_iso_*/*U*_eq_	Occ. (<1)
O1	−0.444 (3)	0.8211 (8)	0.6197 (8)	0.067 (3)	0.65 (3)
C1	−0.573 (3)	0.8426 (17)	0.7583 (10)	0.096 (4)	0.65 (3)
H1A	−0.720338	0.841550	0.802321	0.144*	0.65 (3)
H1B	−0.426110	0.779408	0.785013	0.144*	0.65 (3)
H1C	−0.497179	0.923524	0.737445	0.144*	0.65 (3)
C2	−0.688 (2)	0.8170 (10)	0.6822 (7)	0.0457 (19)	0.65 (3)
H2A	−0.835749	0.880321	0.653798	0.055*	0.65 (3)
H2B	−0.764188	0.735211	0.701695	0.055*	0.65 (3)
O1'	−0.490 (3)	0.8195 (11)	0.6094 (9)	0.031 (2)	0.35 (3)
C1'	−0.563 (4)	0.864 (2)	0.7540 (12)	0.055 (4)	0.35 (3)
H1A'	−0.678101	0.843919	0.808005	0.083*	0.35 (3)
H1B'	−0.368939	0.840230	0.769233	0.083*	0.35 (3)
H1C'	−0.585013	0.953027	0.722506	0.083*	0.35 (3)
C2'	−0.652 (6)	0.796 (3)	0.6957 (15)	0.064 (5)	0.35 (3)
H2A'	−0.849502	0.819587	0.682424	0.077*	0.35 (3)
H2B'	−0.633848	0.707008	0.729060	0.077*	0.35 (3)
C3	−0.3156 (5)	0.7161 (2)	0.61512 (16)	0.0395 (6)	
C4	−0.1133 (5)	0.7365 (2)	0.53762 (15)	0.0360 (5)	
C5	0.0617 (6)	0.6365 (3)	0.53244 (17)	0.0510 (7)	
H5	0.052119	0.558702	0.577298	0.061*	
C6	0.2516 (6)	0.6501 (3)	0.46139 (17)	0.0517 (7)	
H6	0.368657	0.581337	0.458992	0.062*	
C7	0.2706 (5)	0.7644 (2)	0.39378 (15)	0.0352 (5)	
C8	0.0976 (6)	0.8652 (2)	0.39978 (18)	0.0502 (7)	
H8	0.110938	0.943380	0.355718	0.060*	
C9	−0.0955 (6)	0.8518 (2)	0.47027 (18)	0.0475 (7)	
H9	−0.213650	0.920337	0.472518	0.057*	
C10	0.5352 (5)	0.6934 (2)	0.29175 (15)	0.0375 (5)	
H10	0.443589	0.619824	0.316787	0.045*	
C11	0.7498 (4)	0.6990 (2)	0.22091 (14)	0.0338 (5)	
C12	0.8139 (5)	0.5934 (2)	0.19733 (15)	0.0364 (5)	
H12	0.719860	0.521090	0.225004	0.044*	
C13	1.0217 (5)	0.5963 (2)	0.13136 (15)	0.0360 (5)	
C14	1.1643 (5)	0.7032 (2)	0.08737 (15)	0.0355 (5)	
C15	1.0965 (5)	0.8105 (2)	0.11319 (14)	0.0342 (5)	
C16	0.8937 (5)	0.8077 (2)	0.17850 (15)	0.0353 (5)	
H16	0.850675	0.878663	0.194993	0.042*	
C17	1.2144 (6)	1.0171 (3)	0.09638 (19)	0.0529 (7)	
H17A	1.023098	1.052175	0.086809	0.079*	
H17B	1.340280	1.078339	0.061842	0.079*	
H17C	1.255566	0.993340	0.159176	0.079*	
N1	0.4682 (4)	0.78390 (19)	0.32058 (13)	0.0379 (5)	
N2	1.0868 (5)	0.4819 (2)	0.11024 (15)	0.0501 (6)	
O2	−0.3425 (5)	0.61540 (19)	0.67179 (14)	0.0630 (6)	
O3	0.931 (7)	0.396 (2)	0.138 (2)	0.065 (4)	0.64 (12)
O4	1.297 (6)	0.4818 (19)	0.061 (2)	0.068 (4)	0.64 (12)
O3'	0.979 (12)	0.387 (3)	0.157 (4)	0.063 (7)	0.36 (12)
O4'	1.259 (8)	0.475 (3)	0.050 (2)	0.062 (5)	0.36 (12)
O5	1.3628 (4)	0.71443 (18)	0.02210 (12)	0.0485 (5)	
H5A	1.380 (7)	0.637 (3)	0.016 (2)	0.066 (10)*	
O6	1.2501 (4)	0.90927 (16)	0.06865 (12)	0.0452 (5)	

### Atomic displacement parameters (Å^2^)

**Table:**

	*U*^11^	*U*^22^	*U*^33^	*U*^12^	*U*^13^	*U*^23^
O1	0.079 (5)	0.058 (3)	0.074 (5)	−0.024 (3)	0.050 (4)	−0.036 (3)
C1	0.125 (8)	0.095 (7)	0.088 (7)	−0.070 (6)	0.036 (6)	−0.048 (5)
C2	0.049 (3)	0.037 (3)	0.049 (3)	−0.007 (2)	0.021 (3)	−0.014 (2)
O1'	0.031 (4)	0.026 (4)	0.030 (4)	0.000 (3)	0.007 (3)	−0.005 (3)
C1'	0.064 (7)	0.066 (8)	0.044 (7)	−0.001 (7)	0.024 (6)	−0.034 (5)
C2'	0.072 (8)	0.061 (7)	0.061 (7)	−0.009 (6)	0.037 (6)	−0.027 (6)
C3	0.0415 (13)	0.0415 (13)	0.0378 (13)	−0.0099 (10)	0.0089 (10)	−0.0160 (11)
C4	0.0380 (12)	0.0384 (12)	0.0344 (12)	−0.0077 (9)	0.0082 (10)	−0.0158 (10)
C5	0.0599 (17)	0.0441 (14)	0.0375 (13)	0.0080 (12)	0.0107 (12)	−0.0027 (11)
C6	0.0563 (16)	0.0460 (14)	0.0403 (14)	0.0194 (12)	0.0108 (12)	−0.0047 (11)
C7	0.0304 (11)	0.0434 (13)	0.0326 (11)	−0.0037 (9)	0.0058 (9)	−0.0143 (10)
C8	0.0608 (17)	0.0346 (12)	0.0506 (15)	−0.0049 (11)	0.0260 (13)	−0.0116 (11)
C9	0.0523 (15)	0.0356 (12)	0.0531 (15)	−0.0022 (11)	0.0217 (12)	−0.0160 (11)
C10	0.0329 (12)	0.0426 (13)	0.0349 (12)	−0.0024 (9)	0.0080 (9)	−0.0114 (10)
C11	0.0287 (11)	0.0413 (12)	0.0293 (11)	0.0005 (9)	0.0045 (9)	−0.0101 (9)
C12	0.0362 (12)	0.0373 (12)	0.0319 (11)	−0.0038 (9)	0.0089 (9)	−0.0075 (9)
C13	0.0390 (12)	0.0364 (12)	0.0322 (11)	−0.0003 (9)	0.0070 (9)	−0.0123 (9)
C14	0.0310 (11)	0.0443 (13)	0.0298 (11)	−0.0031 (9)	0.0070 (9)	−0.0116 (10)
C15	0.0328 (11)	0.0396 (12)	0.0293 (11)	−0.0060 (9)	0.0023 (9)	−0.0100 (9)
C16	0.0335 (11)	0.0405 (12)	0.0330 (11)	−0.0009 (9)	0.0020 (9)	−0.0146 (10)
C17	0.0652 (18)	0.0472 (15)	0.0487 (15)	−0.0164 (13)	0.0091 (13)	−0.0175 (12)
N1	0.0330 (10)	0.0450 (11)	0.0355 (10)	−0.0024 (8)	0.0090 (8)	−0.0144 (9)
N2	0.0594 (14)	0.0436 (12)	0.0463 (13)	−0.0056 (10)	0.0210 (11)	−0.0163 (10)
O2	0.0736 (14)	0.0533 (12)	0.0516 (12)	−0.0069 (10)	0.0272 (10)	−0.0067 (9)
O3	0.091 (8)	0.052 (4)	0.058 (7)	−0.029 (5)	0.032 (6)	−0.024 (4)
O4	0.070 (6)	0.050 (4)	0.090 (8)	−0.009 (4)	0.042 (6)	−0.038 (4)
O3'	0.087 (12)	0.043 (5)	0.062 (13)	−0.018 (6)	0.034 (10)	−0.022 (6)
O4'	0.076 (9)	0.066 (9)	0.054 (9)	−0.022 (6)	0.038 (6)	−0.036 (5)
O5	0.0520 (11)	0.0497 (11)	0.0465 (10)	−0.0119 (8)	0.0250 (8)	−0.0211 (9)
O6	0.0486 (10)	0.0433 (9)	0.0445 (10)	−0.0126 (8)	0.0152 (8)	−0.0156 (8)

### Geometric parameters (Å, º)

**Table:**

O1—C3	1.314 (8)	C8—C9	1.383 (3)
O1—C2	1.481 (8)	C8—H8	0.9300
C1—C2	1.464 (12)	C9—H9	0.9300
C1—H1A	0.9600	C10—N1	1.267 (3)
C1—H1B	0.9600	C10—C11	1.465 (3)
C1—H1C	0.9600	C10—H10	0.9300
C2—H2A	0.9700	C11—C12	1.375 (3)
C2—H2B	0.9700	C11—C16	1.406 (3)
O1'—C3	1.359 (10)	C12—C13	1.394 (3)
O1'—C2'	1.484 (13)	C12—H12	0.9300
C1'—C2'	1.476 (17)	C13—C14	1.392 (3)
C1'—H1A'	0.9600	C13—N2	1.450 (3)
C1'—H1B'	0.9600	C14—O5	1.343 (3)
C1'—H1C'	0.9600	C14—C15	1.415 (3)
C2'—H2A'	0.9700	C15—O6	1.357 (3)
C2'—H2B'	0.9700	C15—C16	1.370 (3)
C3—O2	1.203 (3)	C16—H16	0.9300
C3—C4	1.485 (3)	C17—O6	1.425 (3)
C4—C5	1.373 (3)	C17—H17A	0.9600
C4—C9	1.386 (3)	C17—H17B	0.9600
C5—C6	1.380 (3)	C17—H17C	0.9600
C5—H5	0.9300	N2—O3'	1.220 (13)
C6—C7	1.381 (3)	N2—O3	1.220 (9)
C6—H6	0.9300	N2—O4	1.239 (9)
C7—C8	1.378 (3)	N2—O4'	1.239 (12)
C7—N1	1.422 (3)	O5—H5A	0.91 (4)
			
C3—O1—C2	119.9 (8)	C7—C8—C9	121.1 (2)
C2—C1—H1A	109.5	C7—C8—H8	119.5
C2—C1—H1B	109.5	C9—C8—H8	119.5
H1A—C1—H1B	109.5	C8—C9—C4	120.2 (2)
C2—C1—H1C	109.5	C8—C9—H9	119.9
H1A—C1—H1C	109.5	C4—C9—H9	119.9
H1B—C1—H1C	109.5	N1—C10—C11	123.2 (2)
C1—C2—O1	104.4 (10)	N1—C10—H10	118.4
C1—C2—H2A	110.9	C11—C10—H10	118.4
O1—C2—H2A	110.9	C12—C11—C16	119.85 (19)
C1—C2—H2B	110.9	C12—C11—C10	118.3 (2)
O1—C2—H2B	110.9	C16—C11—C10	121.8 (2)
H2A—C2—H2B	108.9	C11—C12—C13	119.1 (2)
C3—O1'—C2'	108.6 (15)	C11—C12—H12	120.5
C2'—C1'—H1A'	109.5	C13—C12—H12	120.5
C2'—C1'—H1B'	109.5	C14—C13—C12	122.3 (2)
H1A'—C1'—H1B'	109.5	C14—C13—N2	120.63 (19)
C2'—C1'—H1C'	109.5	C12—C13—N2	117.1 (2)
H1A'—C1'—H1C'	109.5	O5—C14—C13	125.9 (2)
H1B'—C1'—H1C'	109.5	O5—C14—C15	116.3 (2)
C1'—C2'—O1'	115.1 (16)	C13—C14—C15	117.70 (19)
C1'—C2'—H2A'	108.5	O6—C15—C16	125.9 (2)
O1'—C2'—H2A'	108.5	O6—C15—C14	113.86 (18)
C1'—C2'—H2B'	108.5	C16—C15—C14	120.2 (2)
O1'—C2'—H2B'	108.5	C15—C16—C11	120.9 (2)
H2A'—C2'—H2B'	107.5	C15—C16—H16	119.5
O2—C3—O1	123.4 (5)	C11—C16—H16	119.5
O2—C3—O1'	123.2 (6)	O6—C17—H17A	109.5
O2—C3—C4	123.5 (2)	O6—C17—H17B	109.5
O1—C3—C4	112.8 (5)	H17A—C17—H17B	109.5
O1'—C3—C4	112.8 (6)	O6—C17—H17C	109.5
C5—C4—C9	118.9 (2)	H17A—C17—H17C	109.5
C5—C4—C3	118.4 (2)	H17B—C17—H17C	109.5
C9—C4—C3	122.8 (2)	C10—N1—C7	118.1 (2)
C4—C5—C6	120.7 (2)	O3—N2—O4	125 (2)
C4—C5—H5	119.7	O3'—N2—O4'	119 (3)
C6—C5—H5	119.7	O3'—N2—C13	118 (2)
C5—C6—C7	121.0 (2)	O3—N2—C13	119.8 (14)
C5—C6—H6	119.5	O4—N2—C13	115.5 (13)
C7—C6—H6	119.5	O4'—N2—C13	122.7 (19)
C8—C7—C6	118.3 (2)	C14—O5—H5A	103 (2)
C8—C7—N1	118.7 (2)	C15—O6—C17	117.55 (18)
C6—C7—N1	123.0 (2)		
			
C3—O1—C2—C1	102.5 (12)	C11—C12—C13—N2	178.6 (2)
C3—O1'—C2'—C1'	106 (2)	C12—C13—C14—O5	−178.7 (2)
C2—O1—C3—O2	−16.7 (18)	N2—C13—C14—O5	1.8 (4)
C2—O1—C3—C4	168.9 (11)	C12—C13—C14—C15	1.4 (4)
C2'—O1'—C3—O2	16.1 (18)	N2—C13—C14—C15	−178.1 (2)
C2'—O1'—C3—C4	−171.3 (13)	O5—C14—C15—O6	−1.5 (3)
O2—C3—C4—C5	−3.1 (4)	C13—C14—C15—O6	178.4 (2)
O1—C3—C4—C5	171.2 (8)	O5—C14—C15—C16	179.2 (2)
O1'—C3—C4—C5	−175.7 (8)	C13—C14—C15—C16	−0.9 (3)
O2—C3—C4—C9	176.5 (3)	O6—C15—C16—C11	−179.2 (2)
O1—C3—C4—C9	−9.2 (9)	C14—C15—C16—C11	0.0 (3)
O1'—C3—C4—C9	3.9 (8)	C12—C11—C16—C15	0.5 (3)
C9—C4—C5—C6	−0.2 (4)	C10—C11—C16—C15	179.1 (2)
C3—C4—C5—C6	179.4 (3)	C11—C10—N1—C7	−175.8 (2)
C4—C5—C6—C7	0.0 (5)	C8—C7—N1—C10	−149.9 (3)
C5—C6—C7—C8	1.0 (4)	C6—C7—N1—C10	32.9 (4)
C5—C6—C7—N1	178.2 (3)	C14—C13—N2—O3'	172 (4)
C6—C7—C8—C9	−1.7 (4)	C12—C13—N2—O3'	−7 (4)
N1—C7—C8—C9	−179.1 (2)	C14—C13—N2—O3	−168 (2)
C7—C8—C9—C4	1.5 (4)	C12—C13—N2—O3	12 (2)
C5—C4—C9—C8	−0.5 (4)	C14—C13—N2—O4	9 (2)
C3—C4—C9—C8	179.9 (3)	C12—C13—N2—O4	−170 (2)
N1—C10—C11—C12	177.4 (2)	C14—C13—N2—O4'	−4 (3)
N1—C10—C11—C16	−1.3 (4)	C12—C13—N2—O4'	177 (3)
C16—C11—C12—C13	0.0 (3)	C16—C15—O6—C17	5.0 (3)
C10—C11—C12—C13	−178.7 (2)	C14—C15—O6—C17	−174.3 (2)
C11—C12—C13—C14	−1.0 (4)		

### Hydrogen-bond geometry (Å, º)

**Table:**

*D*—H···*A*	*D*—H	H···*A*	*D*···*A*	*D*—H···*A*
O5—H5*A*···O4	0.91 (4)	1.73 (4)	2.54 (2)	146 (3)
O5—H5*A*···O4^i^	0.91 (4)	2.49 (4)	3.23 (3)	138 (3)
C12—H12···O2^ii^	0.93	2.60	3.471 (3)	156

Symmetry codes: (i) −*x*+3, −*y*+1, −*z*; (ii) −*x*, −*y*+1, −*z*+1.
